# RT-PCR-based assessment of the SD Bioline Rota/Adeno Antigen-based test in infants with and without diarrhea

**DOI:** 10.1186/s12985-023-01999-z

**Published:** 2023-03-03

**Authors:** Gédéon Prince Manouana, Paul Alvyn Nguema-Moure, Alexandru Tomazatos, Moustapha Nzamba Maloum, C.-Thomas Bock, Peter G. Kremsner, Thirumalaisamy P. Velavan, Akim Ayola Adegnika, Sandra Niendorf

**Affiliations:** 1 Institute of Tropical Medicine, University of Tübingen, Tübingen, Germany; 2 Centre de Recherches Médicales de Lambaréné, Lambaréné, Gabon; 3 Department Infectious Disease Epidemiology, Bernhard Nocht Institute for Tropical Medicine, Hamburg, Germany; 4 Department of Infectious Diseases, Robert Koch Institute, Berlin, Germany; 5 German Center for Infection Research (DZIF), Tübingen, Germany; 6 Vietnamese-German Center for Medical Research (VG-CARE), Hanoi, Vietnam; 7 Fondation Pour La Recherche Scientifique, Cotonou, Bénin

**Keywords:** Rotavirus A, SD BIOLINE Rota/Adeno Ag RDT, RT-qPCR, Diagnostic

## Abstract

**Background:**

Rotavirus A (RVA) infections remain a major cause of severe acute diarrhea affecting children worldwide. To date, rapid diagnostic tests (RDT) are widely used to detect RVA. However, paediatricians question whether the RDT can still detect the virus accurately. Therefore, this study aimed to evaluate the performance of the rapid rotavirus test in comparison to the one-step RT-qPCR method.

**Methods:**

A cross-sectional study was conducted in Lambaréné, Gabon, from April 2018 to November 2019. Stool samples were collected from children under 5 years of age with diarrhoea or a history of diarrhoea within the last 24 h, and from asymptomatic children from the same communities. All stool samples were processed and analysed using the SD BIOLINE Rota/Adeno Ag RDT against a quantitative reverse transcription PCR (RT-qPCR), which is considered the gold standard.

**Results:**

For a total of 218 collected stool samples, the overall sensitivity of the RDT was 46.46% (confidence interval (CI) 36.38–56.77), with a specificity of 96.64% (CI 91.62–99.08) compared to one-step RT-qPCR. After confirming the presence or absence of RVA gastroenteritis, the RDT showed suitable results in detecting rotavirus A-associated disease, with a 91% concordance with the RT-qPCR. Furthermore, the performance of this test varied when correlated with seasonality, symptoms, and rotavirus genotype.

**Conclusion:**

This RDT showed high sensitivity and was suitable for the detection of RVA in patients with RVA gastroenteritis, although some asymptomatic RVA shedding was missed by RT-qPCR. It could be a useful diagnostic tool, especially in low-income countries.

## Background

Diarrheal diseases remain one of the major causes of morbidity and mortality in children under five years, causing over 500,000 annual deaths worldwide, particularly in developing countries. One of the main causative pathogens of gastroenteritis in children is the group A rotavirus (RVA) [1] (family *Reoviridae*). The non-enveloped, triple-layered viral particle contains a genome of eleven double-stranded RNA segments (dsRNA) that code for six structural (VP1-VP4, VP6, VP7) and six non-structural (NSP1-NSP6) proteins [2]. Of these segments, VP7 (glycoprotein or G) and VP4 (spike protease-sensitive or P) are two outer capsid proteins determining the RVA G/P genotypes [3]. In addition to determining the G/P types of rotaviruses, characterization of the viruses by whole genome sequencing of all eleven segments is beneficial to better understand the important role of reassortants, in the introduction of new RVA variants into the human population [4]. Globally, the most common and widespread strains infecting humans are G1P[8], G2P[4], G3P[8], G4P[8], and G9P[8] and G12P[8] [5].

In 2016, an estimated 128,500 children worldwide died from RVA-associated disease [6]. Hence, rapid and accurate diagnosis of RVA is important for the treatment of individual patients, and subsequent management, as well as for facilitating population-based screening programmes for effective prevention.

Several diagnostic techniques exist for the detection of rotaviruses. Electron microscopy was first used for the detection of RVA virus particles. However, this technique is not routinely available due to its high cost, required expertise and low sensitivity [7]. Since the 1980s, enzyme-linked immunosorbent assays (ELISA) have been widely used and their results are considered satisfactory compared to electron microscopic results [7–9]. Recently, molecular techniques such as PCR have replaced other diagnostic tests with the advantage of higher sensitivity and specificity [10]. However, these techniques require expensive equipment and advanced technical skills, which hinders on-site disease testing in developing countries [11]. In addition, RT-PCR detects up to 14% more rotavirus infections, compared to ELISA in a healthy control group, suggesting that in some RT-PCR-positive infectious disease cases, rotavirus A may not actually be the cause of the disease [12].

In this context, several simple and inexpensive rapid immunochromatographic diagnostic tests (RDTs) are now commercially available. These RDTs do not require extensive training of the reader or specialized laboratory equipment. Hence, they seem particularly useful in resource-constrained healthcare centres in low and middle-income countries, where such tests may improve patient management and community-based screening programmes. However, their performance remains controversial, and the obtained results may remain doubtful in asymptomatic subjects due to low viral loads. Furthermore, only few studies have investigated their validity in tropical countries, such as those conducted by Ope et al. [13] and Thangjui et al*.* [14]. The present study (i) determined the correlation between RDT results and RVA viral load, as assessed by cycle threshold (Ct) values; (ii) assessed the diagnostic accuracy of the Bioline Rota/Adeno SD RDT and (iii) determined the effect of sampling period on RDT results.

## Methods

### Study samples

Stool samples were collected and tested for RVA by quantitative RT-PCR and subsequently sequenced for molecular surveillance of RVA in Gabonese children under five years of age residing in Lambaréné, Gabon and the surrounding rural region between April 2018 and November 2019 [15]. Children with diarrhoea or a history of diarrhoea within the last 24 h were recruited from the outpatient department of two main hospitals (Hôspital Albert Schweitzer and Centre Hospitalier Régional Georges Rawiri de Lambaréné). Stool samples were then randomly collected from asymptomatic children of the same age who lived in the same neighborhoods and thus had the same living conditions as the symptomatic children. Of the samples selected on the basis of the RDT analysis performed, the predominant strains were G12P[6] (n = 20), G1P[8] (n = 19), and G8P[8] (n = 14) among the RVA RT-qPCR positives [15].

### Laboratory procedures

#### SD BIOLINE Rota/Adeno Ag RDT processing

Fresh stool samples were tested using a commercially available SD BIOLINE Rota/Adeno Ag RDT (Standard Diagnostics INC, Kyonggi, Korea). A small amount of stool sample was transferred into the diluent provided by the kit manufacturer using a swab. The suspension was homogenized by swirling (at least ten times). Four drops of diluted faecal material were dispensed into the circular window of the test card. The test results were read after 20 min as instructed by manufacturer.

#### RNA isolation, rotavirus detection and genotyping

Viral RNA was extracted from 140 µl of the stool suspension in RNAlater using the QIAamp viral RNA Mini Kit (QIAGEN, Hilden, Germany). All steps of the RNA isolation were performed following the manufacturer’s instructions. The genomic RNA was eluted in a total volume of 60 µl and stored at – 80 °C.

The presence of RVA was first confirmed using a single-step RT-qPCR protocol targeting the NSP4 gene, as described previously [16]. Briefly, RNA extracts were first diluted and denaturated at 95 °C for 1 min, then RT-qPCR was performed in a total volume of 12 µl using the SuperScript III/Platinum Taq OneStep kit (Invitrogen, Carlsbad, CA). The reaction mix contained PCR buffer, forward primer (RoA 25-s) 5′-GCTTTTAAAAGTTCTGTTCCGAG, and reverse primer (RoA 26a-as) 5′-ACTCAATGTGTAGTTGAGGTCGG, probe 5′‐VIC‐ATCTTTCCGCACGC‐MGB and SuperScript III/Platinum Enzyme mix under the following condition: 15 min 50 °C, 2 min 95 °C, followed by 45 cycles of 15 s 94 °C, 32 s 55 °C (acquisition). Samples with a cycle threshold (Ct) ≤ 39 were considered positive.

All RVA positive samples were subsequently genotyped based on the amplification of the VP7 and VP4 genes, as previously described [15].

#### Definition of asymptomatic or symptomatic cases due to RVA infection

A cut-off value of Ct = 24 was proposed after evaluating the ability of RT-qPCR to distinguish between the presence and absence of RVA gastroenteritis in children under 5 years of age using enzyme immunoassay as the gold standard [17, 18].

### Statistical analysis

Statistical analysis was performed using GraphPad Prism 6.00. Sensitivity, specificity, positive predictive value (PPV) and negative predictive value (NPV) were calculated to assess the performance of the SD BIOLINE Rota/Adeno Ag RDT in comparison to the RT-qPCR defined as the reference method. The test performance measures are presented as percentages with their respective 95% confidence interval (CI). Ct value distribution between diarrhoea cases and asymptomatic controls was tested using the Mann Whitney test and we performed Chi-squared tests and a two-sided *p*-value of less than 0.05 was considered statistically significant.

## Results

**Fig. 1:**
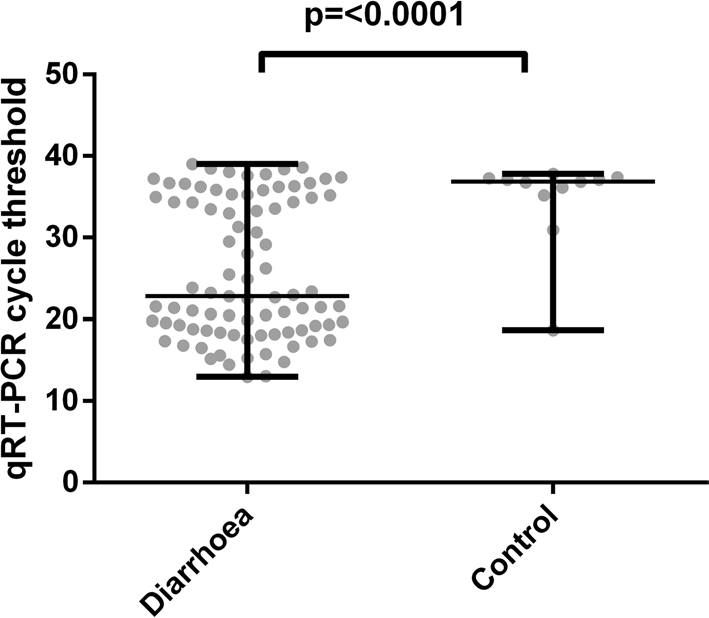
Distribution of RT-qPCR Ct values between diarrhoeal cases and community controls. The median (IQR) for patients was 22.80 (12.99–39.01), and for asymptomatic children, it was 36.85 (18.70 to 37.83). The distribution of Ct values between the two groups was statistically significant (*p* < 0.0001)

Of the 218 stool samples analysed, 72% (156/218) were symptomatic children with a median age of twelve months, while the median age of healthy children was 24 months. Of the total population, 58% (126/218) were male and 62% (136/218) lived in semi-urban areas (Lambaréné). Overall, 45% (99/218) of the selected samples were positive for RVA. There was a significant difference in distribution of Ct values between diarrhoea cases (median = 22.80; interquartile range:12.99–39.01) and community controls (median = 36.85; interquartile range:18.70 to 37.83) (Fig. 1). Among the 99 RVA-positive samples, we observed a bimodal distribution of Ct values in symptomatic children showing two distinct groups, one with low Ct values (n = 47; Ct range: 12.99–23.87) and another with high Ct values (n = 38; Ct range: 24.94–39.01). Among community controls, only one sample was detected with low Ct value out of the 14 RVA positive by RT-qPCR. The SD BIOLINE Rota/Adeno Ag RDT was able to detect 46% (46/99) of RVA-RT-qPCR-positive samples. Overall, the sensitivity and specificity values of the Rota/Adeno Ag RDT for RVA detection in RT-qPCR positive samples were 46.46% and 96.64%, yielding positive and negative predictive values of 92% and 68.45%, respectively (Table 1).

**Table 1:** Diagnostic characteristics of SD Bioline Rota/Adeno Rapid test

RDT result	Number of specimens		Sensitivity (95% CI)	Specificity (95% CI)	PPV (95% CI)	NPV (95% CI)
	RT-qPCR positive	RT-qPCR negative				
Positive	46	4	46.46 (36.38–56.77)	96.64 (91.62–99.08)	92 (80.77–97.78)	68.45 (60.85–75.39)
Negative	53	115				

Of the 99 samples with a positive RT-qPCR result for RVA, 14 were from community controls and had no positive RDT results. When comparing RT-qPCR and RDT results for RVA according to diarrhoea status, the sensitivity and specificity values of the Rota/Adeno Ag RDT for RVA detection were 0% and 97.92% in healthy children, respectively, while the sensitivity and specificity were 54.12% and 95.77% in children with diarrhoea (Table 2).

**Table 2:** Diagnostic characteristics of SD Bioline Rota/Adeno Rapid test according to diarrhoea status

RDT result	Number of specimens		Sensitivity (95% CI)	Specificity (95% CI)	PPV (95% CI)	NPV (95% CI)
	RT-qPCR positive	RT-qPCR negative				
No Diarrhoea						
Positive	0	1	0.0 (0.0–23.16)	97.92 (88.93–99.95)	0.0 (0.0–97.50)	77.07 (64.50–86.85)
Negative	14	47				
Diarrhoea						
Positive	46	3	54.12 (42.96–64.98)	95.77 (88.14–99.12)	93.88 (83.13–98.72)	63.55 (53.69–72.64)
Negative	39	68				

In the patient’s group (n = 85), 47 samples (55.3%) showed a low Ct value below 24. Of these, 43 samples (91%) were tested positive with RDT. Within the healthy control group (n = 14), one sample was identified with a low Ct value, which tested negative with RDT (Table 3).

**Table 3:** Correlation between rotavirus viral load and antigen test performance

Ct-value *	RT-qPCR positive samples (n)	SD Bioline RDT n (%)
Patients		
Low	47	43 (91)
High	38	3 (8)
Community controls		
Low	1	0 (0)
High	13	0 (0)

*Low (Ct < 24); High (Ct > 24) [17, 18]

Among the predominant RVA genotypes identified in the recently published study [15], overall, the rapid test detected 15 positive samples for G12P[6], followed by 13 and nine positive samples for G8P[8] and G1P[8], respectively (Table 4). When comparing RT-qPCR and RDT results for detection of the these genotypes, RT-qPCR identified more than RDT, showing a concordance of 93% (13/14), 75% (15/20) and 47% (9/19) for G8P[8], G12P[6] and G1P[8], respectively. When comparing RT-qPCR and RDT results for the detection of these genotypes among the RVA gastroenteritis cases, we found 100% concordance for G8P[8] and G12P[6], and 82% concordance for G1P[8]. Of note, G3P[6] and G3+G12P[6] were detected by RDT with 100% concordance either among all RVA positive samples or among RVA gastroenteritis cases. Furthermore, the RDT identified almost all genotypes, with the exception of G12P[8], the triple G mixed types (G1+G8+G12P[8]) and the partial G/P mixed types, which showed a high/low Ct value of 36.24 and 22.7, respectively.

**Table 4:** Correlation between rotavirus genotypes and antigen detection test performance

	All RVA positive samples		RVA gastroenteritis	
RVA genotypes	RT-qPCR (n)	SD Bioline RDT [n (%)]	RT-qPCR (n)	SD Bioline RDT [n (%)]
G8P[8]	14	13 (93)	12	12 (100)
G3P[8]	5	5 (100)	5	5 (100)
G12P[6]	20	15 (75)	15	15 (100)
G9P[8]	2	1 (50)	2	1 (50)
G1P[8]	19	9 (47)	11	9 (82)
G12P[8]	2	0 (0)	2	0 (0)
G3+G12P[6]	2	2 (100)	1	1 (100)
G1+G8+G12P[8]	1	0 (0)	0	0 (0)
Partial G/P mixed types*	7	0 (0)	1	0 (0)
Untypeable	27	1 (4)	1	0 (0)

*GxP[8]; G8P[x]; GxP[6]; G1P[x]; G3P[x]; G3+G8P[x]; G8+G12P[x]; G3P[x]

**Fig. 2:**
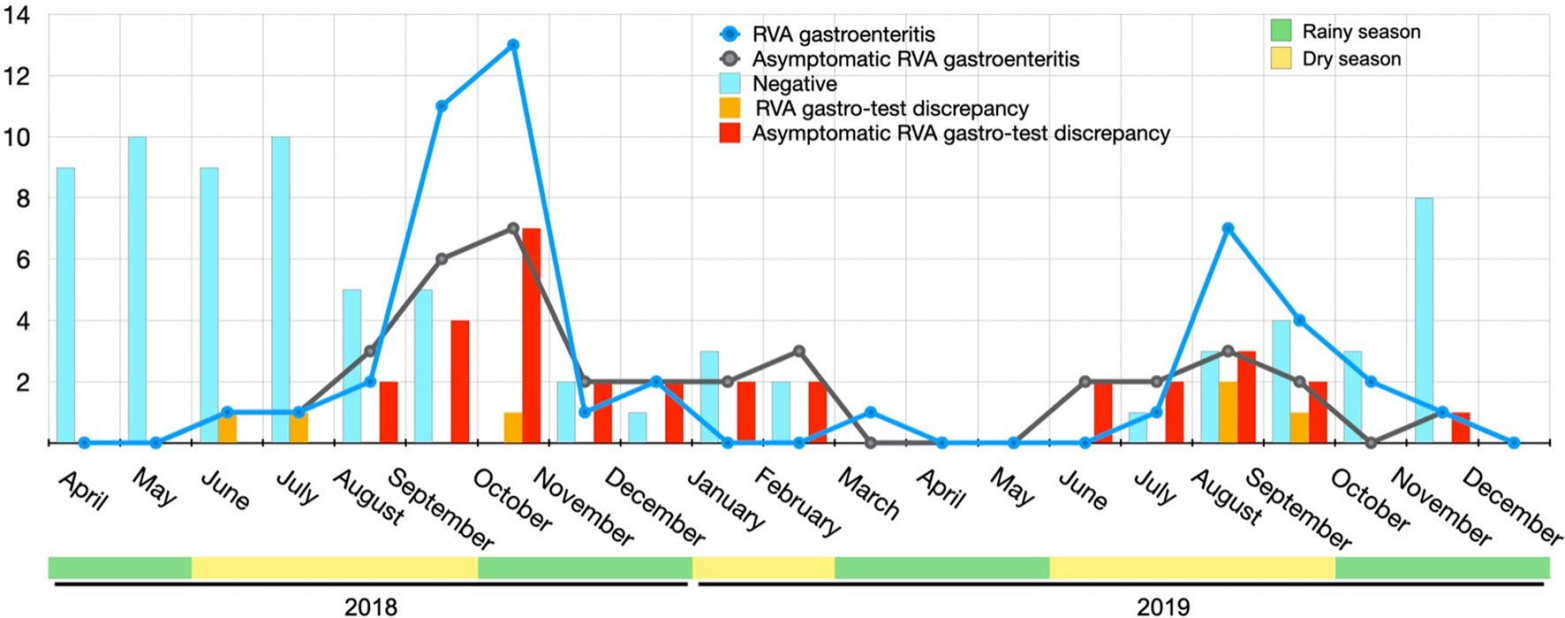
Seasonal burden of RVA gastroenteritis among Gabonese children under the age of five years from Lambaréné and discrepancies between RT-qPCR and RDT results for the detection of RVA during the sampling period (April 2018–December 2019)

Most RVA infections were recorded during the dry seasons with peaks in September and October 2018 (turn of the seasons) and September 2019, period in which slightly more cases of RVA gastroenteritis occurred compared to asymptomatic RVA infection. Discrepancies between RT-qPCR and RDT were observed for the detection of both RVA gastroenteritis and asymptomatic RVA infection during the dry seasons, with a few cases of asymptomatic infection during the rainy seasons. These discrepancies were RDT false negatives when detecting RVA infections (Fig. 2). In addition, we examined the performance of the RDT across the sampling period or seasons. Overall, the sensitivity and specificity of the RDT to detect RVA-RT-qPCR positive samples were slightly higher during the cumulative rainy seasons compared to the cumulative dry seasons during the study (Table 5).

**Table 5:** Diagnostic characteristics of SD Bioline Rota/Adeno Rapid test according to the seasons (only for the patients’ group)

RDT result	Number of specimens		Sensitivity (95% CI)	Specificity (95% CI)	PPV (95% CI)	NPV (95% CI)
	RT-qPCR positive	RT-qPCR negative				
Cumulated rainy seasons						
Positive	19	1	57.58 (39.22–74.52)	97.06 (84.67–99.93)	95 (75.13–99.87)	70.21 (55.11–82.66)
Negative	14	33				
Cumulated dry seasons						
Positive	27	2	51.92 (37.63–65.99)	94.59 (81.81–99.34)	93.1 (77.23–99.15)	58.33 (44.88–70.93)
Negative	25	35				

Cumulated rainy seasons: April–May 2018 + October-December 2018 + March–May 2019 + October–November 2019

Cumulated dry seasons: June–September 2018 + January–February 2019 + June–September 2019

## Discussion

In developing countries, where the infection is most prevalent and consequential, there is a great need for effective diagnostic methods for RVA. In the search for alternative methods to electron microscopy and advanced molecular tools, we assessed the diagnostic accuracy of commercially available SD BIOLINE Rota/Adeno Ag RDT. This point-of-care test, with its high-performance parameters (sensibility: 100% and specificity: 99.7%) according to the manufacturer, requires no refrigeration or advanced technical knowledge or skills, is time-saving and easy to perform. However, our results on RDT performance characteristics showed a weak sensitivity (46.46%), while specificity was higher (96.64%) for the detection of RVA-RT-qPCR positive samples. The observed specificity suggests that this RDT can reliably detect samples lacking RVA. In contrast, the sensitivity determined in this study suggests that the assay performs poorly in detecting all RVA RT-qPCR positive samples. The observed overall sensitivity is lower than that reported by the manufacturer and by Thweesak Chieochansin [19], in which the authors reported sensitivity and specificity of 93.75% and 96.17%, respectively. However, when considering high viral loads or RVA-associated diarrhoea, we found that a positive RVA RDT result was strongly associated with diarrhoea and a low Ct value (91% concordance, Table 3), whereas only three asymptomatic RVA infections were identified by RDT. This observation allowed us to conclude that this RDT is very well suited to detect symptomatic gastroenteritis associated with RVA. These findings are consistent with data from the United Kingdom and the United States, suggesting that enzyme immunoassays are the best method for diagnosing infectious intestinal disease associated with RVA [18, 20]. Furthermore, our findings could also suggest that RT-qPCR-positive and RDT-negative samples do not reflect rotavirus disease as described elsewhere [21].

The RDT showed no sensitivity among community controls (mean Ct = 34.66 for positive cases), while the sensitivity was 54.12% in children with diarrhoea (mean Ct = 25.40) (Table 2). In addition, low sensitivity of this RDT was also observed for RVA detection in asymptomatic children (Table 3). These data indicate a close correlation between RDT performance and viral load. Furthermore, they reinforce the hypothesis that the clinical implications of a positive RT-qPCR result are uncertain, as RT-qPCR can detect fecal shedding of RVA in up to 30% of asymptomatic children [18, 22]. In addition, our findings showed overall low sensitivities of RDT compared to RT-qPCR depending on the season. The RDT showed decreased sensitivity during the cumulative dry seasons (Table 4). The same observation was made in a previous study [18] in which the sensitivity was lower during the season of low RVA prevalence. These results could be correlated with the fluctuation of temperatures during the sampling period, which may influence the seasonality of RVA infection incidence. Thus, it was observed (Fig. 2) that the ratio of number of patients with RVA-associated gastroenteritis over to the number of patients who are positive but have no RVA-associated gastroenteritis (Ct > 24) changes during the season.

Furthermore, the bimodal distribution of Ct values observed in this study may be related to heterogeneous host response to infection affecting viral replication, as well as the timing of sampling relative to the moment of infection (i.e., longitudinal sampling of host would elucidate that). Ct values are semiquantitative measurements commonly used for identifying higher clinical severity or transmissibility, although Ct and viral titers do not correlate closely in the case of clinical severity for some pathogens (e.g., SARS-CoV-2). Because we identified only RVA and AGE may have other/additional aetiological agents (e.g. norovirus), Ct values can be affected by co-infection [23–25].

This SD BIOLINE rapid test was able to detect different genotypes of RVA. Although some false negatives were observed when detecting asymptomatic RVA infections, this RDT successfully detected most of the genotypes. Interestingly, it was even able to detect non-typeable RVA strain. Table 4 shows that one third of the 99 RVA isolates were only partially or not typeable. Thus, more work has to be done on these isolates before attempting to correlate RVA genotype with performance in RDTs. Our results thus suggest that the non-detection of some genotypes may be due to low viral load. This is the case for G1P[8], which has a detection rate of 47% among all samples positive by RT-qPCR, and 82% for symptomatic samples. This concurs with the report of Tate and collaborators [20], who point out that when the virus was detected only by RT-qPCR, the genotypes could not be determined, probably due to the low level of viral shedding.

The lack of exclusion of adenovirus as an aetiological agent of gastroenteritis is a limitation of this study. In addition, of the 38 asymptomatic cases of RVA gastroenteritis, eleven were also positive for noroviruses, astroviruses or sapoviruses [26]. Therefore, we cannot exclude the possibility that other pathogens could be responsible for the patients' symptoms in this study. Moreover, lack of data such as temperature and humidity might be a missed opportunity to better assess the findings in a way which could assist the improvement of RDT’s performance.

## Conclusions

In conclusion, the overall diagnostic accuracy of the SD BIOLINE Rota/Adeno Ag RDT is low for the detection of RVA compared to RT-qPCR. However, this test showed a good diagnostic accuracy for the detection of RVA in patients with acute gastroenteritis. The inability to identify asymptomatic individuals may raise concerns about their use in national control programmes to prevent transmission of RVA. In such a case, a molecular method such as RT-qPCR should be considered as it has a higher sensitivity in detecting low viral loads.

## Data Availability

All data generated or analysed in this study are included in this publication.

## Abbreviations

RVA: Rotavirus A
RDT: Rapid diagnostic tests
RT: Reverse transcriptase
qPCR: Quantitative polymerase chain reaction
CI: Confidence interval
NSP: Non-structural protein
ELISA: Enzyme-linked immunosorbent assay
Ct: Cycle threshold
min: Minute
PPV: Positive predictive value
NPV: Negative predictive value
